# New Evidence for *Cotinus coggygria* Scop. Extracts Application in Gastrointestinal Ailments

**DOI:** 10.3390/ph18010098

**Published:** 2025-01-15

**Authors:** Dejan Stojković, Nina Dragičević, Marija Ivanov, Nevena Gajović, Milena Jurišević, Ivan Jovanović, Marina Tomović, Jelena Živković

**Affiliations:** 1 Institute for Biological Research “Siniša Stanković”—National Institute of the Republic of Serbia, University of Belgrade, Bulevar despota Stefana 142, 11108 Belgrade, Serbia; marija.smiljkovic@ibiss.bg.ac.rs; 2 Department of Pharmacy, Singidunum University, Danijelova 32, 11000 Belgrade, Serbia; ndragicevic@singidunum.ac.rs; 3 Center for Molecular Medicine and Stem Cell Research, Faculty of Medical Sciences, University of Kragujevac, Svetozara Markovica 69, 34000 Kragujevac, Serbia; gajovicnevena@yahoo.com (N.G.); milena.jurisevic13@gmail.com (M.J.); ivanjovanovic77@gmail.com (I.J.); marinapopo@gmail.com (M.T.); 4 Department of Pharmacy, Faculty of Medical Sciences, University of Kragujevac, Svetozara Markovica 69, 34000 Kragujevac, Serbia; 5 Institute for Medicinal Plants Research “Dr. Josif Pančić”, Tadeuša Košćuška 1, 11000 Belgrade, Serbia

**Keywords:** *Cotinus coggygria*, flavonoids, phenolic acids, gastrointestinal, antimicrobial, cytotoxic

## Abstract

Background/Objectives: *Cotinus coggygria* Scop. is traditionally used for treatment of various gastrointestinal ailments. In this study, we investigated the phytochemical profile and biological activities of leaves, bark and flowers extracts of *C. coggygria.* Methods: Phytochemical analysis was performed using HPLC. The antimicrobial activity of water and methanolic extracts of *C. coggygria* leaves, bark and flowers towards various oral fungal and bacterial strains of clinical origin was tested by use of a microdilution assay. Additionally, their cytotoxic activity was determined against different gastrointestinal carcinoma cell lines (CAL27, FaDu, SW480, HCT116 and MRC-5) in concentrations ranging from 3.125 to 400 µg/mL for 48 and 72 h. Results: The presence of numerous flavonoid and phenolic compounds such as sulfuretin, gallic acid, rutin, hyperoside, and isoquercitrine was detected. *Micrococcus luteus*, *Streptococcus parasanguinis*, and *Candida tropicalis* were the most sensitive microbiological species, with MICs of 0.12 mg/mL for the most effective extracts. Additionally, the cytotoxic activity of the samples against different gastrointestinal carcinoma cell lines (CAL27, FaDu, SW480, HCT116, and MRC-5) was determined in concentrations ranging from 3.125 to 400 µg/mL. Among the tested samples, the methanolic leaf extract exhibited the highest cytotoxic capacity, and the possible mechanism could be related to its inhibitory effect on the release of proinflammatory cytokine in CD4+ cells. Conclusions: The traditional use of *C. coggygria* for gastrointestinal diseases may be substantiated by its ability to inhibit the growth of harmful microorganisms and its promising cytotoxic properties. The methanolic leaf and flower extracts show significant potential for future clinical applications, and further studies are warranted to explore their mechanisms and applications in medical treatments.

## 1. Introduction

Infectious disease and malignancy are recognized as leading causes of morbidity and mortality worldwide [1]. Almost 20% of human malignancies can be related to infectious agents, and chronic infection is presumed to be a significant contributor to the initiation of several cancer types. Also, inflammation can elevate cancer progression and stimulate the process of invasion and metastasis. Although previous studies confirmed the link among certain bacteria and cancer, there is as yet no clear understanding of its mechanism [2].

Oral cavity infections may present one of the more prominent preventable causes of cancer [3]. Cancers of the gastrointestinal tract are a significant health problem and represent almost 20% of all cancer-related deaths in both men and women [4]. The intestinal tract is one of the largest immune organs in the body, with gut-associated lymphoid tissue, and it contains lymphocytes, plasma cells, and macrophages, which produce mediators such as cytokines [5]. The incidence rate of gastrointestinal cancers is very high, and most patients are diagnosed at the late stage of disease and lack effective treatment [6].

In recent decades, the emphasis on identifying therapeutic plant-based active principles has led to significant advancements in the identification and use of natural compounds to treat various diseases. Numerous traditionally used plant species have been investigated in order to establish their potential application in the prevention or treatment of oral diseases. Extracts from the leaves, twigs, wood, and inflorescences of *Cotinus coggygria* Scop. (smoketree) are used in the ethnomedicine of Eastern and Southeastern Europe and China as antidiarrhoetic, anti-inflammatory and antiparadentosis agents [7], as well as for the treatment of gastric and duodenal ulcer [8]. As an anti-inflammatory agent, it is mainly used for the treatment of skin injuries and mucosal tissues (buccal, gastric and intestinal). These indications are mainly in correlation with the high content of tannins and essential oils. In previous studies, a broad spectrum of pharmacological activities has been shown for extracts of various *C. coggygria* parts (leaves, flowers, heartwood, and bark).

Our study was undertaken to investigate the antimicrobial effects of water and methanolic extracts of *C. coggygria* leaves, bark and flowers on various oral fungal and bacterial strains of clinical origin, as well as their cytotoxic activity against different gastrointestinal carcinoma cell lines. The presented manuscript provides exclusive data on smoketree’s biological activities, which may expand its utilization.

## 2. Results and Discussion

### 2.1. Phytochemical Analysis

A large number of phytoconstituents have been previously isolated from different smoketree plant parts. Studies have mainly shown that flavonoids are the most important and abundant group of biologically active constituents of *Cotinus* species, followed by phenolic acids [9,10]. According to our study, sulfuretin was the dominant compound in bark extracts, while gallic acid prevailed in flower and leaf extracts (Table 1). Also, sulfuretin and fisetin, which were found in higher amounts in bark extracts, were present only in traces in leaf extracts, while in flower extracts these compounds were not detected. Regarding flavonoid compounds, isoquercetin was dominant in all of the investigated samples. The UV spectra of compounds identified in *Cotinus* extracts, as well as chromatograms of *C. coggygria* water leaf, flower and bark extracts, are presented in Supplementary Material. The obtained results are in line with previous studies. Namely, Antal et al. [11] showed that sulfuretin was the major compound in stems and branches of *C. coggygria*, representing 0.38–0.69% of the extract, depending on the sample. Having in mind flower and leaf extracts, Savikin et al. [12] proved that gallic acid and its derivatives are dominant.

According to our results, while discussing the type of extraction solvent, a higher content of gallic acid was obtained using water as the extraction solvent, whereas higher contents of sulfuretin, fisetin and rutin derivatives were found in methanolic extracts.

The results of the phytochemical analysis show that the investigated extracts are enriched with biologically active compounds. However, data on the pharmacological effects of different smoketree extracts are less available.

### 2.2. Antifungal and Antibacterial Activities of Tested Extracts

The oral cavity is home to a diverse range of bacterial microbiota, which may play a role in maintaining microbial homeostasis. Disruptions to this balance could potentially contribute to dysbiosis, which has been suggested as a factor in the development of various conditions, including carcinoma [13]. For example, Tateda et al. [14] found *Streptococcus anginosus* to be highly abundant in gingival smears of patients with head and neck squamous cell carcinoma.

*Candida* is regularly present in the oral microbiome of healthy individuals. On the other hand, it may cause mild or severe disease in immunocompromised patients. High-dose corticosteroid therapy, as well as a broad-spectrum antibacterial therapy, may cause mucocutaneous infections in the GI tract, such as oral candidiasis [15]. There are reports that *Candida* species are indirectly connected with the carcinogenesis of various forms of cancer, such as oral squamous cell carcinoma [16].

In Table 2, the anticandidal potentials of six extracts that were tested for antifungal activity against different *Candida* species are presented. The most susceptible fungal species was *C. tropicalis* ATCC 750, with MIC 0.12 mg/mL for the CCBM extract and MIC 0.25 mg/mL for CCLM, CCFW and CCLW extracts. *C. tropicalis* has emerged as one of the most important *Candida* species in terms of epidemiology and virulence [17]. It is regarded as a significant agent of candidemia, especially in neoplasia patients [18]. The tested extracts also showed promising antifungal capacity towards *C. albicans* and *C. krusei*, as indicated by the MIC values of 0.25 to 4 mg/mL. *C. auris* CDC B11903 was the most resistant among the tested species, with MIC ranging 1–8 mg/mL, and it was most susceptible to the treatment with extracts CCBM, CCFM and CCLW (MIC 1 mg/mL). This pathogen has numerous virulence qualities, and shows multi-drug resistance patterns to common antifungal therapies used for other invasive *Candida* infections [19]. Considering individual compounds, San et al. [20] showed that gallic acid was highly active against *C. albicans* and *C. tropicalis* with an MIC value 9.8 µg/mL for both fungal strains. Yang et al. [21] showed the antifungal activity of fisetin against *Hypocrea nigrigans*, which was higher compared to the activity of the antifungal compound cycloheximide that was used as the control. In the same study, both fisetin and gallic acid exhibited strong activities against *Trichoderma virens*, which were higher compared to applied cyclohexamide.

The antibacterial activity of the *C. coggygria* plant species was evaluated previously against different bacterial strains such as *Staphylococcus aureus*, *Bacillus subtilis*, *Klebsiella pneumoniae*, *Escherichia coli*, *Micrococcus lysodeicticus*, Pseudomonas aeruginosa and Enterococcus faecalis [9,22]. Matić et al. [9] showed that the bacteria most sensitive toward water extracts of *C. coggygria* leaf were *E. coli* and *M. lysodeikticus*. Tunc et al. [22] reported the highest activity of *C. coggygria* leaf water extract against *E. faecalis*, while the methanolic extract of the same plant part was the most active against *S. aureus*. However, there is little evidence regarding the bactericidal effects of extracts obtained from different smoketree parts on clinical microorganisms. The antibacterial activities of the extracts tested in our study are provided in Table 3. They exhibited antibacterial activities against all tested strains, but at different levels. The most potent antibacterial potential could be observed for the CCLM and CCFM extracts, with MIC values 0.12 mg/mL displayed towards the majority of the tested bacterial strains. The most susceptible strains were *M. luteus* and *S. parasanguinis*. On the other hand, the most resistant bacterial species tested was *E. cloacae* (ot_18), with an MIC up to 8 mg/mL, with CCLW extract being the most active towards this species. Streptomycin, when used as a positive control, consistently exhibited potent antibacterial activity, and none of the investigated extracts revealed stronger activity. The MIC values for streptomycin were 3-fold higher compared to the most active extracts, at 0.12 mg/mL.

There is a significant difference in the effects of aqueous and methanolic extracts of smoketree flowers. Namely, the aqueous extract showed greater activity against most of the tested bacterial strains. This can be explained with reference to the high content of gallic acid. According to Sorrentino et al. [23], gallic acid and its esters, which are also characteristic of the *C. coggygria* plant species, are capable of inducing irreversible changes in the membranes of microbial cells, leading to consequent rupturing or pore formation and the leakage of essential intracellular contents. In a study conducted by Sen et al. [20], they identified a moderate antibacterial activity against *S. aureus* and *S. epidermidis,* with MIC values of 78 and 156 µg/mL. In summary, CCBM and CCFW extracts have demonstrated consistent efficacy against both fungi and bacteria, displaying relatively lower MIC and MBC/MFC values. Further studies may be warranted to explore the potential therapeutic applications of these extracts, considering the observed variations in activity against different microbial strains. To the best of our knowledge, this is the first report related to the antimicrobial effects of *C. coggygria* plant parts against the clinical bacterial and fungal strains used in this study.

### 2.3. Cytotoxic Capacity of C. coggygria Extracts

**Figure 1:**
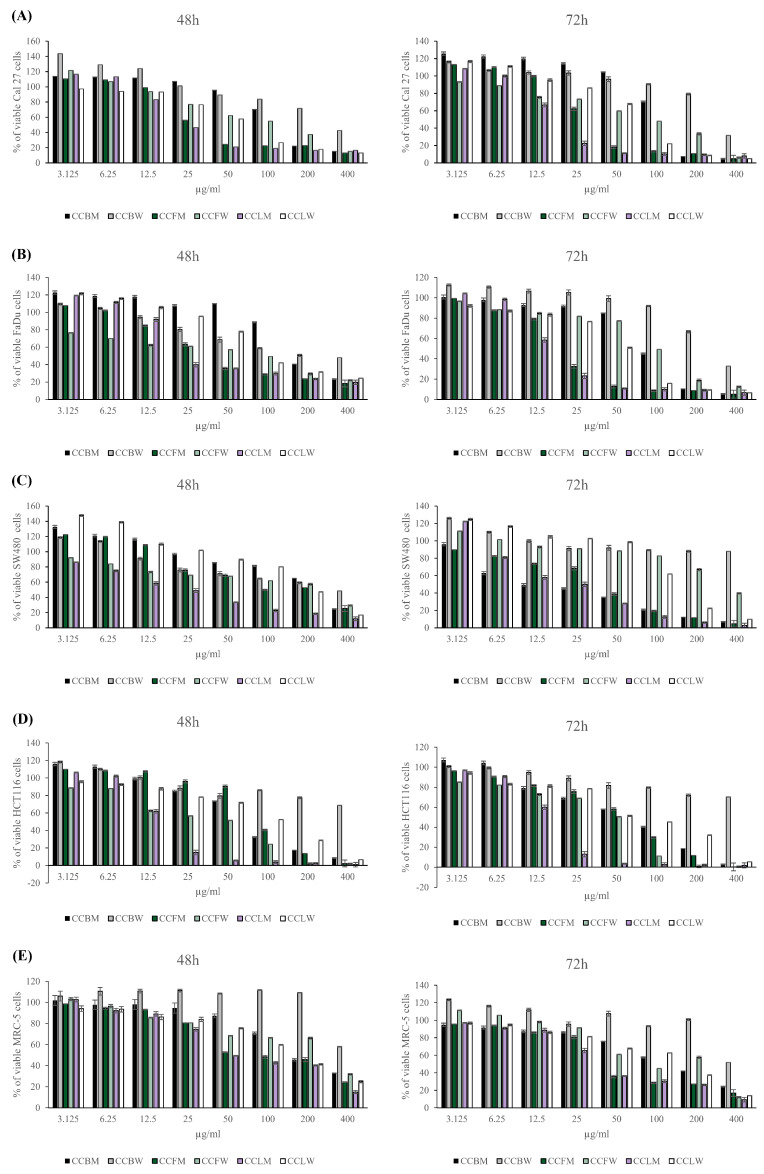
Cytotoxic effect of *C. coggygria* extracts against human squamous cell carcinoma and human colon cancer cell lines. (**A**) Effects of *C. coggygria* extracts on CAL27 (**A**), FaDu (**B**), SW480 (**C**), HCT116 (**D**) and MRC-5 (**E**) cell viability after 48 and 72 h, determined by MTT assay. The results represent the mean ± SD of three independent experiments (each performed in triplicates).

In order to determine the antiproliferative potential of *C. coggygria* bark, flower and leaf methanolic and water extracts, MTT assays were performed. Human squamous cell carcinoma cell lines (tongue, CAL27 and pharynx FaDu) and human colon cancer cell lines (SW480 and HCT116) were treated with *C. coggygria* methanolic and water extracts in a concentration range of 3.125–400 µg/mL for 48 and 72 h. The human fibroblast cell line (MRC-5) was also used as a control, for noncancerous cells. Among the tested concentrations, all *C. coggygria* extracts reduced the viability of cancer cell lines in a dose-dependent manner (Figure 1). For *C. coggygria* bark (CCBM), flower (CCFM) and leaf (CCLM) methanolic extracts, better cytotoxic capacities were observed, especially when in higher concentrations, compared to their corresponding water extracts (CCBW, CCFW and CCLW). Namely, the IC_50_ values for water extracts (CCBW, CCFW and CCLW) were 1.1- to 3-fold higher compared to those of methanolic extracts (CCBM, CCFM and CCLM) (Table 4). CCBW was the least toxic to all tested cancer cells, with IC_50_ values greater than 300 µg/mL (Table 4). The higher cytotoxic activity of CCBM compared to CCBW could be due to the content of flavonoid compounds fisetin and sulphuretin. Yan et al. demonstrated that fisetin treatment inhibited the growth of gastric carcinoma cells by suppressing ERK 1/2 activation [24]. At the same time, fisetin suppressed tumor growth and metastasis in gastric cancer, suggesting its potential for therapeutic application [25]. Sulfuretin showed significant effects in various cancer models due to its cytotoxic, anti-inflammatory, and apoptotic properties [26,27]. However, specific studies on gastric carcinoma are limited. Most of the available evidence points to the anticancer activity of fisetin, and since sulfuretin is a sulfated derivative of fisetin, it may share similar mechanisms of action. Human squamous cell carcinoma and colon cancer cell lines appeared to be most sensitive to the CCLM extract, with IC_50_ values ranging from 17.0 µg/mL (HCT116 cells after 72 h treatment) to 28.6 µg/mL (SW 480 cells after 48 h treatment) (Table 4).

The extract of CCFM exhibited similar cytotoxic activity against CAL27 (IC_50_ 33.3 µg/mL after 48 h) and FaDu (IC_50_ 24.3 µg/mL after 72 h) compared to CCLM (IC_50_ 28.1 µg/mL and 22.7 µg/mL, respectively). Interestingly, the observed reduction in MRC-5 cells’ viability after extract treatments for 48 h and 72 h indicate that noncancerous cells were less sensitive to the cytotoxic effect of *C. coggygria*, with an IC_50_ from 0.5- to 2-fold higher compared to cancer cells (Figure 1, Table 4). The highest selectivity indexes against HCT116 cells corresponded to CCFM (SI 8.63 after 48 h and SI 3.96 after 72 h treatment) and CCLM (Selective Index (SI) 3.03 after 48 h treatment), while those against SW480 cells corresponded to CCBM (SI 7 after 72 h treatment) and those against FaDu cells corresponded to CCFW (SI 3.75 after 48 h treatment), CCLM (SI 3.03 after 48 h treatment) and CCLW (SI 3.32 after 72 h treatment) (Table 5). Due to its promising cytotoxic capacity and higher SI than the other three, the CCLM extract was considered suitable for the further investigation of its possible mechanism of action.

Recently, the cytotoxic effects of *C. coggygria* were investigated by other researchers. The cytotoxic effects of the methanolic extracts of the leaves and flowers of the *C. coggygria* were first confirmed on the human cervical (HeLa) and colon (LS174) carcinoma cell lines [12]. The tumoricidal capacity of *C. coggygria* leaves ethanolic extract was confirmed on human breast cancer cell lines MCF7 and T47D, cervical carcinoma HeLa, ovarian carcinoma A2780 and squamous cell carcinoma A431, with a modest selectivity index [10,28,29]. The cytotoxic effect of *C. coggygria* leaf extract was also observed against Hep3B liver cancer cells [30]. Considering individual compounds, Li et al. [31] showed that fisetin represents a potential therapeutic strategy for treating human oral squamous cell carcinoma, modulating proliferation by blocking p21-activated kinase 4 (PAK 4) signaling pathways. Farsad-Naeimi et al. [32] demonstrated that the application of 100 mg of fisetin per day improved the inflammatory status in patients with colorectal cancer by reducing the IL-8 levels in plasma. Our in vitro results demonstrate that methanolic *C. coggygria* bark (CCBM), flower (CCFM) and leaf (CCLM), as well as the corresponding water extracts (CCBW, CCFW and CCLW), inhibited the proliferation of human squamous cell carcinoma and human colon cancer cells in a dose-depended manner (Figure 1, Table 4). Methanolic extracts (CCBM, CCFM, and CCLM) more efficiently inhibited the growth of CAL27, FaDu, SW480 and HCT116 cells compared to the corresponding water extracts (CCBW, CCFW and CCLW) (Figure 1, Table 4). CCBW was recognized as the least toxic to all examined cancer and noncancerous cell lines. In addition, the highest cytotoxic capacity was shown by CCLM. The cytotoxic effect of CCFM against CAL27 and Fadu cells was comparable to the cytotoxic capacity of CCLM. Both extracts contained gallic acid, hyperoside and isoquercetine as the dominant compounds. These can be identified as partially responsible for the exhibited activity. Gallic acid can induce apoptosis in HeLa cells by activating intrinsic and extrinsic pathways. This includes the upregulation of pro-apoptotic proteins like Bax and the downregulation of anti-apoptotic proteins like Bcl-2 [33]. At the same time, in HCT116 cells, gallic acid can trigger apoptosis by disrupting mitochondrial membrane potential, which leads to the activation of caspases (especially caspase-3 and caspase-9), essential mediators of the intrinsic apoptotic pathway [34]. Rutin derivatives, hyperoside and isoquercetine, can initiate apoptosis through the mitochondrial pathway, which involves mitochondrial membrane depolarization and cytochrome c release. These flavonoids can alter the Bax/Bcl-2 protein expression ratio, upregulating the pro-apoptotic protein Bax and downregulating the anti-apoptotic protein Bcl-2, thus contributing to mitochondrial dysfunction and cell death [35]. Nevertheless, considering that these compounds were also present in other tested extracts, it can be concluded that classes of compounds other than polyphenolics contributed to the observed effects in our study. Additionally, the specific ratio of these compounds (gallic acid, hyperoside and isoquercetine) may be significant for the exhibited activity. A study conducted by Khafif et al. showed that the combination of different polyphenolic compounds (e.g., epigallocatechin gallate (EGCG), and curcumin) exhibited synergistic effects, resulting in enhanced anticancer activity in normal, premalignant and malignant human oral epithelial cells [36]. According to them, the specific combination ratio of these compounds played a significant role in their activity.

Impressively, in our study, the tested extracts, except CCBW and CCBM, more efficiently inhibited the growth of CAL27, FaDu, SW480 and HCT116 cancer cells than MRC-5, human fibroblast cells (Figure 1, Table 4 and Table 5). The indiscriminate action of numerous cytotoxic agents is the main reason unsuccessful treatment due to various side effects. For this reason, the selectivity of the investigated compounds towards tumor cells compared to non-tumor cells is being emphasized increasingly often.

### 2.4. CCLM Facilitates Apoptotic Cell Death in Human Colon Cancer Cells (HCT116)

**Figure 2:**
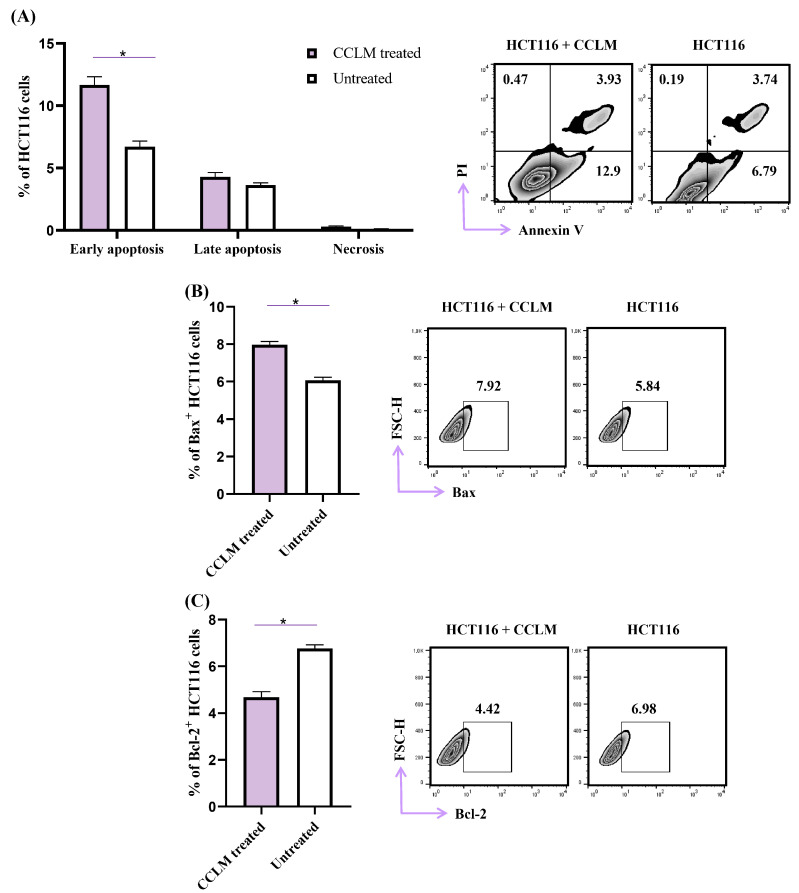
In the presence of CCLM, human colon cancer cells (HCT116) underwent apoptosis. (**A**) Apoptosis of CCLM (20 μg/mL)-treated and -non-treated HCT116 cells was analyzed by flow cytometry after double staining with Annexin V (FITC) and PI. The data are presented as means ± SD (*n* = 3), * *p* < 0.05 (Mann–Whitney U test), followed by representative dot plots. The analysis of Bax (**B**) and Bcl-2 (**C**) expression in HCT116 cells preexposed to CCLM (20 μg/mL) for 48 h using flow cytometry. Data are presented as mean ± SD. * *p* < 0.05 (Mann–Whitney U test).

In order to understand in greater detail, the background of CCLM’s cytotoxic capacity, human colorectal cancer cells, HCT116, were exposed to CCLM (20 µg/mL) for 48 h, and the analysis of phosphatidylserine externalization using double staining with Annexin-V and Propidium Iodide was performed. Pharmacologically untreated HCT116 cells served as the control. As shown in Figure 2, CCLM treatment increased the percentage of early apoptotic AnnV^+^PI^−^ HCT 116 cells (11.7% ± 1.2) compared to untreated cells (6.7% ± 0.9) (*p* = 0.016; Figure 2A). After CCLM treatment, the percentage of late apoptotic AnnV^+^PI^+^HCT 116 cells (4.3% ± 0.8), as well as necrotic AnnV^−^PI^+^HCT 116 cells (0.3% ± 0.1), remained unchanged in comparison to untreated cells (3.6% ± 0.4, 0.12% ± 0.1 respectively) (Figure 2A). As Bcl-2 family proteins are amongst the key molecules in the regulation of apoptotic cell death [37], further impacts of CCLM treatment on the expression of pro-apoptotic Bax and anti-apoptotic Bcl-2 protein were observed. A significant increment in Bax^+^ HCT 116 cells was observed after CCLM treatment (7.9% ± 0.4), compared to control cells (6.0% ± 0.4) (*p* = 0.008; Figure 2B). Also, CCLM treatment decreased the percentage of Bcl-2^+^ HCT 116 cells (4.7% ± 0.6) in comparison to untreated cells (6.8% ± 0.3) (*p* = 0.008; Figure 2C). These results indicate that after CCLM treatment, human colon carcinoma cells underwent apoptosis.

### 2.5. CCLM Induced Cell Cycle Arrest in G0/G1 Phase

**Figure 3:**
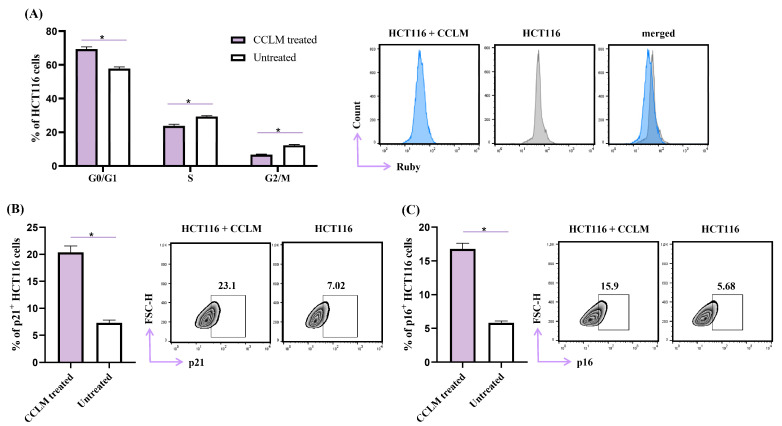
CCLM induced arrest at the G0/G1 checkpoint of the HCT116 cell cycle. (**A**) The HCT116 cell cycle was analyzed by flow cytometry after preincubation with CCLM (20 μg/mL) for 48 h. Data are presented as the mean ± SD, * *p* < 0.05 (Mann–Whitney U test). Analysis of p21 (**B**) and p16 (**C**) expression in HCT116 cells exposed to CCLM (20 μg/mL) for 48 h using flow cytometry. Data are presented as mean ± SD. * *p* < 0.05 (Mann–Whitney U test).

The HCT116 cell cycle profile was determined after 48 h of exposure to CCLM (20 µg/mL) (Figure 3A). CCLM treatment increased the percentage of HCT116 cells in the G0/G1 phase (57.8% for untreated vs. 69.4% for CCLM treated HCT116 cells). The percentages of CCLM-treated cells in the S and in G2/M phases decreased, respectively (S phase, untreated 29.3% vs. CCLM-treated cells 23.7%; G2/M phase, untreated 12.2% vs. CCLM-treated 6.7%) (Figure 3A). The expression of CDK inhibitors p21 and p16 was determined in continuation. After exposure to CCLM, the percentage of p21^+^ HCT116 cells (20.4% ± 2.9) was increased compared to control cells (7.3% ± 1.2) (Figure 3B). A similar pattern was observed for p16^+^ HCT116 cells treated with CCLM (16.7% ± 1.1 vs. untreated cells 5.8% ± 0.7) (Figure 3C).

Due to the fact that p21 is a major inhibitor of the cell cycle [38], these results demonstrate that the inhibition of HCT116 cell proliferation in the presence of CCLM was mainly caused by arresting cells in the G0/G1 phase and modulating levels of CDK.

The equilibrium among cell proliferation and apoptosis is crucial for unhindered cell growth [39]. As CCLM induced apoptosis, the next step was to analyze the potential antiproliferative effects of CCLM by flow cytometry. After CCLM treatment, cell cycle progression was interrupted, and the HCT116 cells were arrested in the G0/G1 phase (Figure 3). Choi et al. reported that isoquercetin (the dominant compound in CCLM extract) effectively inhibited the proliferation of benign prostatic hyperplasia (BPH-1) cells cells by inducing cell cycle arrest in the G0/G1 phase [40]. The study also showed that isoquercetin suppressed the PI3K/Akt/mTOR signaling pathway, and promotes cell survival and proliferation.

Cell cycle regulation is tightly regulated by cyclins, cyclin-dependent kinases and CDK inhibitors [41]. CDKs have become target molecules in cancer therapy [42]. Palbociclib is a CDK4/6 inhibitor newly approved for a wide range of cancer therapies, which causes the arrest of the cell cycle in the G1 phase [42,43]. The CDK inhibitor p16 inhibits CDK4/CDK6, while p21 is a pan-CDK inhibitor. The activities of p16 and p21 are linked and vital during G1/S phase transition [38]. CCLM caused a significant increment in INK p16 and CIP/KIP p21 expression in HCT116 cells (Figure 3). This finding indicates that CCLM induced cell cycle arrest at the G0/G1 checkpoint by upregulating the expressions of p16 and p21. It seems that the tumoricidal capacities of CCLM against human colon cancer cells might be achieved in at least two manners: by inhibiting cell proliferation and triggering apoptotic cell death. Based on the calculated selectivity indexes, the tested *C. coggygria* extracts were the carriers of modest selectivity toward cancer cells (Table 5). CCLM was shown to possess the most robust cytotoxic activity against all tested cell lines, with an SI greater than 3 for HCT116 cells. Taking this in account, in order to determine the possible mechanisms of the antitumor effect of the tested *C. coggygria* extracts, human colon cancer cells were exposed to CCLM for 48 h prior to flow cytometry analyses. The obtained results reveal that CCLM facilitates apoptotic cell death and cell cycle arrest at the G0/G1 checkpoint (Figure 2 and Figure 3). In line with our findings, after confirming the cytotoxic effect of the alcoholic extract of *C. coggygria* against a panel of tumor cells (U937, A549, TK6, and MCF7), it was observed that the tumoricidal potential was partially based on the arrest of the cell cycle in the G1 phase [44]. Flavonoids extracted from *C. coggygria* inhibited the proliferation of glioma cancer cells, namely, U87, U251 and DBTRG-05MG [45,46], by triggering the apoptosis of glioblastoma cells via Akt inhibition. Apoptosis is a type of cell death that is preferred in cancer therapy. The pro-apoptotic protein Bax and anti-apoptotic protein Bcl-2 are well established Bcl2 family proteins that regulate the intrinsic, mitochondrial and apoptotic paths [47]. It is known that the overexpression of pro-survival proteins, such as Bcl-2, BCL-XL and Mcl-1, and the downregulation of effectors of apoptosis (Bax, Bak) are tightly linked to the resistance of cancer cells to cytotoxic agents [48]. For this reason, targeting proteins from the Bcl-2 family is becoming one of the leading strategies in the development of new cytotoxic drugs [48]. Our study has demonstrated that CCLM treatment increased the expression of Bax and decreased the expression of Bcl-2 in HCT116 cells, making them more prone to apoptosis (Figure 3).

### 2.6. Effects of CCLM on Functional Phenotype of Th Cells

**Figure 4:**
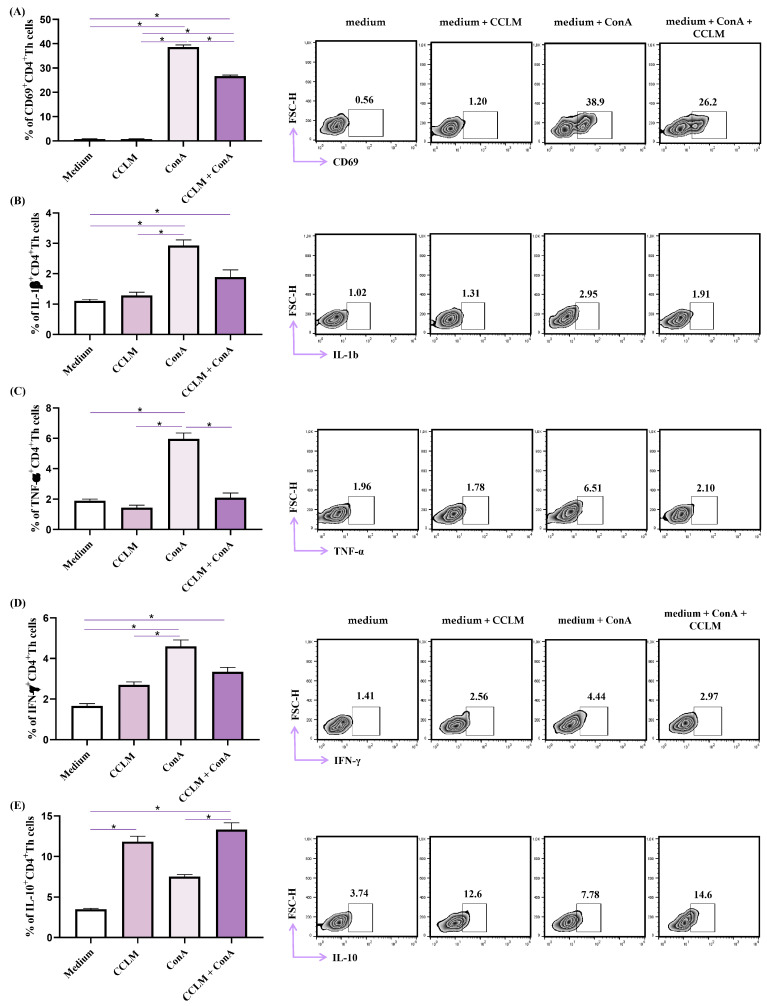
CCLM’s impact on the functional phenotype of Th cells. Splenocytes derived from untreated healthy BALB/C mice were cultivated in medium alone, medium with CCLM (20 μg/mL), medium with ConA (5 μg/mL) or medium with added CCLM (20 μg/mL) and ConA (5 μg/mL) for 24 h. The percentages of CD69+ (**A**), IL-1β+ (**B**), TNF-α+ (**C**), IFN-γ+ (**D**), and IL-10+ (**E**) CD4+ cells were determined by flow cytometry. The data are presented as mean ± SD. Statistical significance was determined by Kruskal–Wallis testing with a post-hoc Mann–Whitney U test * *p* < 0.05.

We further analyzed the phenotypes of CD4^+^ Th cells derived from the spleens of Balb/c mice. ConA stimulation significantly increased the percentage of CD4^+^ cells expressing activation marker CD69, while coincubation with ConA and CCLM decreased CD69 expression, in comparison to cells stimulated with Con-A only (Figure 4, *p* < 0.05). Incubation with CCLM did not affect the expression of CD69. The same phenomenon was observed for proinflammatory cytokines IL-1b, TNF-α and IFN-γ. CCLM did not significantly alter the production of these cytokines in CD4^+^ cells, but ConA significantly increased the percentages of TNF-α- and IFN-γ-producing cells (Figure 4, *p* < 0.05). CCLM together with ConA significantly decreased the percentage of TNF-α-producing cells (Figure 4, *p* < 0.05), while the reductions in IL-1b^+^ and IFN-γ^+^ CD4^+^ cells did not reach statistical significance. ConA, as well as CCLM, increased the production of anti-inflammatory IL-10, while ConA and CCLM together increased its production even further (Figure 4, *p* < 0.05).

Our study demonstrates that CCLM has an inhibitory effect on the release of proinflammatory cytokines in CD4^+^ Th cells derived from the spleens of Balb/c mice. Th cells contribute to acquired immunity and are the most potent producers of cytokines. Taking this into account, we focused on the immunomodulatory effects of CCLM on these cells. Treatment with ConA significantly increased the expression of CD69 and stimulated the production of IL-1β, TNF-α and IFN-γ (Figure 4). The CCLM alone did not affect the production of proinflammatory cytokines, but significantly decreased cytokine production and CD69 expression in ConA-stimulated CD4^+^ Th cells (Figure 4). Finally, CCLM increased the production of anti-inflammatory cytokine IL-10, as did ConA, and had synergistic effects with ConA on IL-10 production (Figure 4). It appears that CCLM has potent inhibitory effects on activated Th cells, by decreasing the expression of activating molecules and the production of proinflammatory cytokines, while it has no effect on inactivated Th cells. Additionally, CCLM stimulates the production of anti-inflammatory IL-10 in the same Th cells. We believe that CCLM has potent anti-inflammatory effects on Th cells. It is not known whether the alteration of the functional phenotype of Th cells by the addition of the extract arises from a nonspecific interaction with the cell membrane or from interaction with a specific receptor. The anti-inflammatory effects of an ethyl acetate fraction of young shoots of *C. coggygria* Scop were reported by Marčetić et al. [49].

## 3. Materials and Methods

### 3.1. Plant Material

Fresh leaves, flowers and bark of *C. coggygria* were collected during May 2023 from a woody habitat near Rogatica in Bosnia (N: 43°79′47.9″, E: 18°99′46.1″). The taxonomic identification and authentication were performed at the Institute for Medicinal Plants Research “Dr Josif Pančić”, and a voucher specimen no. Bot. 202347 (ND) was deposited. The plant material was air-dried prior to extraction.

### 3.2. Preparation of Extracts for Chemical and Biological Activity

Extraction was performed with *C. coggygria* bark, flower and leaf separately, using two different extraction solvents (water and methanol) and water bath extraction at 60 °C with a reflux condenser for two hours. The applied solid to solvent ratio was 1:10. The obtained extracts were further filtered using filter paper, and concentrated at 60 °C using a rotary evaporator in case of the methanolic extracts, while they were freeze-dried for 24 h in the case of the water extracts. The following extracts were obtained: bark methanolic (CCBM), bark water (CCBW), flower methanolic (CCFM), flower water (CCFW), leaf methanolic (CCLM), and leaf water (CCLW). The dried extracts were stored in a vacuum desiccator until being used for further experiments.

### 3.3. HPLC Analysis

Analyses were carried out on an Agilent 1260 RR HPLC instrument (Agilent, Waldbronn, Germany) equipped with a diode-array detector working in the range of 190–550 nm. The samples were separated using the reversed-phase Zorbax SB-C18 (Agilent) analytical column (150 mm × 4.6 mm i.d.; 5 μm particle size). The mobile phase A was 1% *v*/*v* solution of orthophosphoric acid in water, while the mobile phase B was acetonitrile. Gradient elution was performed according to the following scheme: 0–2.6 min, 90% A; 2.6–8 min, 90–85% A; 8–10.8 min, 85% A; 10.8–18 min, 85–80% A; 18–23 min, 80% A; 23–25 min, 80–70% A; 25–27 min, 70–50% A; 27–29 min, 50–20% A; 29–31 min, 20–10% A; 31–34 min, 10–0%; 34–35 min, 0%. The detection wavelengths were set at 260, 280, 320 and 360 nm, and the flow rate was 1 mL/min. The injection volume was 8 μL and the column temperature was maintained at 40 °C. The identification of the compounds was achieved by comparing their UV spectra and retention times with those from commercial standards. The amounts of the compounds were calculated using calibration curves. The results are presented as micrograms per gram of dry weight (μg/g dw) for solid samples, or as miligrams per milliliter (μg/mL) for liquid samples.

### 3.4. Microorganisms and Culture Conditions

The *Candida* species used were clinical isolates *C. krusei* H1/16, *C. glabrata* 4/6/15, *C. albicans* ATCC 10231, *C. tropicalis* ATCC 750, and *C. auris* CDC B 11903, obtained and maintained as described in Ivanov et al. [50].

The following Gram-positive and Gram-negative clinical bacteria were used: *Micrococcus luteus* (dT_9/2), *Rothia mucilaginosa* (oT_22/2), *Streptococcus anginosus* (oT_26), *Streptococcus dysgalactiae* (oT_21/2), *Streptococcus oralis* (oT_5), *Streptococcus parasanguinis* (oT_3), *Streptococcus pyogenes* (dT_14), *Streptococcus salivarius* (dT_12), *Staphylococcus hominis* (oT_14/2), *Enterobacter cloacae* (oT_18), and *Stenotrophomonas maltophilia* (A_12), obtained and maintained as described previously [51].

### 3.5. Anticandidal Activity

The minimal inhibitory and minimal fungicidal concentrations (MIC/MFC) were determined [50]. Fresh overnight *Candida* cultures were adjusted to 1.0 × 10^5^ CFU/well with sterile saline. Microplates with *Candida* and an extract serially diluted in YPD medium were incubated at 37 °C for 24 h, after which the MIC and MFC values were determined. The lowest concentrations that did not induce microscopically observable growth were considered as MIC. For the microscopic determination of growth, an inverted Nikon Eclipse TS2 microscope (Amsterdam, The Netherland) was used, and fungal growth in the wells of 96-well microtiter plates was examined and compared to the control (untreated yeast cells). MFC values were determined as concentrations that did not induce visible fungal growth after the serial sub-cultivation of 10 µL of samples at 37 °C for 24 h. Ketoconazole (SigmaAldrich, Darmstadt, Germany) was used as an antifungal control. Each experiment was performed in triplicate.

### 3.6. Antibacterial Activity

The microdilution method was used in order to determine the minimum inhibitory (MIC) and minimum bactericidal (MBC) concentrations of the examined extracts. Bacteria were adjusted with a spectrophotometer to a final concentration of 1.0 × 10^5^ CFU/well. The extracts were serially diluted in 96-well microtiter plates with Tryptic Soy Broth (TSB) and bacteria and incubated at 37 °C for 24 h. The MIC values of the samples were observed after the addition of iodonitrotetrazolium chloride (INT) (0.2 mg/mL, 40 mL) and incubation at 37 °C for 30 min. The lowest concentration that produced an inhibition of the growth of the bacteria in comparison with the positive control was observed as the MIC. The MBC was determined by the serial sub-cultivation of 10 mL of each well sample into microplates containing 100 mL of the TSB. The lowest concentration that showed no growth after this sub-culturing was determined as the MBC. Streptomycin (SigmaAldrich, Hamburg, Germany) was used as a positive control. Each experiment was performed in triplicate.

### 3.7. Cell Culture

Human squamous cell carcinoma cell lines CAL27 (tongue) and FaDu (pharynx), human colon cancer cell lines (SW480 and HCT116) and human fibroblast cell line MRC-5 were purchased from American Type Culture Collection. The cells were routinely grown, under standard conditions (5% CO_2_, 37 °C), in complete DMEM medium supplemented with 10% fetal bovine serum (FBS), streptomycin, L-glutamine, penicillin and nonessential amino acids (Sigma Aldrich, Munich, Germany).

### 3.8. In Vitro Cytotoxic Assay

Cell growth in the presence of *C. coggyriae* extracts was evaluated by MTT assay as previously reported [52]. All tested cancer cells were seeded in 96-well microplates (5 × 10^3^ cells per well). After 24 h of incubation, the attached cancer cells were exposed to the examined CC extracts (CCBM, CCBW, CCFM, CCFW, CCLM and CCLW) in 2-fold dilution in medium to concentrations ranging from 400 to 3.125 µg/mL for an additional 48 h and 72 h, respectively. After incubation time, MTT solution (5 mg/mL PBS) was added to each well for an additional 4 h. The microplate multimode detector Zenyth 3100 was used in order to determinate optical density (595 nm). Each experiment was performed in triplicates and was repeated three times. The IC_50_ values were calculated based on the results of the MTT assay. The selectivity index (SI) was calculated using the formula: IC_50_ MRC-5/IC_50_ cancer cell line.

### 3.9. Analysis of Cell Death

HCT116 cancer cells were treated with 20 µg/mL *C. coggygria* methanolic extract for 48 h, and control untreated cells were double-stained with Annexin-V and Propidium Iodide as previously described [53]. FACS Calibur Flow Cytometer (BD Biosciences, San Jose, CA, USA) was used in order to conduct flow cytometry and FlowJo software 10.7.2 (Tree Star, Ashland, OH, USA) was used for data analysis by drawing a polygon gate encompassing the target cell population, gating these cells, and subsequently applying gates and statistics to all samples.

### 3.10. Cell Cycle Distribution

The cell cycle was analyzed in HCT116 pre-treated cancer cells and control untreated cells by staining with Vybrant DyeCycle™ Ruby stain, according to the manufacturer’s instructions (Thermo Fisher Scientific, Inc., Waltham, MA, USA). A FACS Calibur Flow Cytometer (BD Biosciences, San Jose, CA, USA) was used, and the data were further analyzed using Flow Jo software (Tree Star).

### 3.11. Flow Cytometry Analysis of Bax, Bcl-2, p16 and p21 Expression

HTC116 cells were pretreated with 20 µg/mL *C. coggygria* leaf methanolic extract for 48 h and then incubated with anti-human Bax monoclonal antibody FITC, anti-human Bcl-2 monoclonal antibody FITC, anti-human p21 monoclonal antibody APC, and rabbit p16 antibody followed by secondary FITC-conjugated Dnk anti-rabbit IgG mAb (Abcam, Cambridge, UK). Flow cytometry was conducted as previously described [54]. The analysis was performed on a FACS Calibur Flow Cytometer (BD Biosciences, San Jose, CA, USA). FlowJo software was used for the data analyses (Tree Star).

### 3.12. Analysis of Functional Phenotype of Th Cells

Freshly isolated splenocytes derived from healthy mice (2 × 10^5^ cells) were cultured for 24 h in supplemented DMEM containing *C. coggygria* leaf methanolic extract (20 µg/mL), concanavalin A (ConA 5 µg/mL), *C. coggygria* extract, and concanavalin A and DMEM medium only. After incubation, the cells were harvested and stained with appropriate antibodies for FACS and evaluated by flow cytometry, as previously described [54]. Fluorochrome-labeled anti-mouse mAbs specifc for CD4, CD69, IL-10, IL-1β, TNF-α, IFN-γ or isotypematched controls (BD Pharmingen, San Diego, CA, USA; Invitrogen, Waltham, MA, USA) were used.

## 4. Conclusions

The traditional use of *C. coggygria* in treating gastrointestinal disorders may be attributed to its antimicrobial properties, which help mitigate the growth of microorganisms associated with gastrointestinal discomfort. Our analysis of the plant’s specialized metabolites through HPLC-DAD have revealed that flavonoids and phenolic acids, particularly sulfuretin and gallic acid, were the dominant compounds present, with variations observed depending on the plant part and extraction solvent used.

The methanolic leaf extract of *C. coggygria* demonstrated promising cytotoxicity and antiproliferative activity, with a selectivity index greater than three, indicating its potential for further pharmacological investigation. Additionally, this extract exhibited anti-inflammatory properties, which may be particularly beneficial in clinical settings where inflammation exacerbates underlying gastrointestinal conditions.

These findings suggest that the methanolic leaf extract of smoketree could offer a multi-target therapeutic approach, combining antimicrobial, cytotoxic, antiproliferative, and anti-inflammatory effects. However, further research is essential to elucidate the precise mechanisms of action underlying its immunomodulatory effects, as well as to assess its safety, efficacy, and potential for clinical application. Continued investigations into the pharmacological profiles of *C. coggygria* could provide valuable insights into its role in managing gastrointestinal diseases and related inflammatory conditions.

## Figures and Tables

**Table 1:** Contents of polyphenolic compounds in tested extracts of *C. coggygria*.

Compound	Extract					
	CCBM	CCBW	CCLM	CCLW	CCFM	CCFW
	(mg/g dw)					
Sulfuretin	164.90	13.58	0.76	0.84	Nd	Nd
Fisetin	22.74	1.98	0.10	0.02	Nd	Nd
Gallic acid	3.17	19.98	2.53	43.29	7.47	77.63
Isoquercetin	5.75	2.73	3.02	6.23	4.71	3.99
Hyperoside	5.67	1.73	1.36	0.63	4.87	2.24
Chlorogenic acid	0.60	0.32	0.38	2.94	0.52	1.82
Quercetin	1.18	0.01	Tr	Tr	0.01	Tr

CCBM—bark methanolic extract, CCBW—bark water extract, CCFM—flower methanolic extract, CCFW—flower water extract, CCLM—leaf methanolic extract and CCLW—leaf water extract, Tr—traces.

**Table 2:** Antifungal activity of extracts; results are in mg/mL.

Strains	CCBM		CCBW		CCFM		CCFW		CCLM		CCLW		Ketoconazole	
	MIC	MFC	MIC	MFC	MIC	MFC	MIC	MFC	MIC	MFC	MIC	MFC	MIC	MFC
*C. albicans* ATCC 10231	1	2	2	4	1	2	1	2	1	2	1	2	0.0016	0.0064
*C. glabrata* 4/6/15	0.5	1	1	2	0.5	1	1	2	2	4	0.5	1	0.0016	0.0064
*C. krusei* H1/16	0.5	1	0.5	1	0.5	1	0.25	0.5	1	2	0.25	0.5	0.0016	0.0032
*C. tropicalis* ATCC 750	0.12	0.25	0.5	1	0.5	1	0.25	0.5	0.25	0.5	0.25	0.5	0.0016	0.0064
*C. auris* CDC B 11903	1	2	2	4	1	2	8	>8	8	>8	1	2	-	-

**Table 3:** Antibacterial activity of extracts; results are in mg/mL.

	CCBM		CCBW		CCFM		CCFW		CCLM		CCLW		Streptomycin	
	MIC	MBC	MIC	MBC	MIC	MBC	MIC	MBC	MIC	MBC	MIC	MBC	MIC	MBC
*Micrococcus luteus* (dT_9/2)	0.25	0.5	0.25	0.5	0.25	0.5	0.12	0.25	0.12	0.25	0.5	1	0.006	0.012
*Rothia mucilaginosa* (oT_22/2)	0.5	1	1	2	0.5	1	0.12	0.25	0.25	0.5	0.25	0.5	0.012	0.025
*Streptococcus anginosus* (oT_26)	0.5	1	0.25	0.5	1	2	0.5	1	0.12	0.25	1	2	0.003	0.006
*Streptococcus dysgalactiae* (oT_21/2)	0.12	0.25	0.25	0.5	0.12	0.25	0.12	0.25	0.25	0.5	0.25	0.5	0.006	0.012
*Streptococcus oralis* (oT_5)	0.5	1	0.5	1	1	2	1	2	0.5	1	1	2	0.012	0.025
*Streptococcus parasanguinis* (oT_3)	0.12	0.25	0.25	0.5	0.5	1	0.12	0.25	0.12	0.25	0.25	0.5	0.003	0.006
*Streptococcus pyogenes* (dT_14)	0.25	0.5	0.25	0.5	2	4	0.12	0.25	0.12	0.25	0.25	0.5	0.003	0.006
*Streptococcus salivarius* (dT_12)	0.5	1	0.5	1	2	4	0.12	0.5	0.25	0.5	0.25	0.5	0.006	0.012
*Staphylococcus hominis* (oT_14/2)	0.5	1	0.25	0.5	1	2	0.12	0.25	0.12	0.25	0.25	0.5	0.038	0.075
*Enterobacter cloacae* (oT_18)	2	4	2	4	2	4	8	>8	8	>8	0.25	0.5	0.038	0.075
*Stenotrophomonas maltophilia* (A_12)	4	8	0.25	0.5	1	2	0.12	0.25	0.12	0.25	0.25	0.5	0.038	0.075

**Table 4:** IC_50_ values for *C. coggygria* extracts against human squamous cell carcinoma cell lines (tongue, CAL27 and pharynx, FaDu), human colon cancer cell lines (SW480 and HCT116) and non-tumor human fibroblast cell line (MRC-5).

Extract	IC_50_ (µg/mL)									
	Cal 27		FADU		SW480		HCT116		MRC-5	
	48 h	72 h	48 h	72 h	48 h	72 h	48 h	72 h	48 h	72 h
CCBM	147.3 ± 9.1 ^$^	122.3 ± 4.5 ^$^	191.2 ± 9.9	75.1 ± 1.1 ^$^	225.5 ± 6.15	20.7 ± 0.2	73.2 ± 1.5	55.8 ± 2.7	196.5 ± 9.1	144.9 ± 8.1
CCBW	325.1 ± 3.8	322.1 ± 12.7	212.4 ± 6.6	306.7 ± 12.4	>400	>400	>400	>400	>400	>400
CCFM	33.3 ± 1.7 ^$^	41.1 ± 0.3 ^$^	37.1 ± 1.4 ^$^	24.3 ± 0.6 ^$^	135.0 ± 9.9	33.15 ± 1.2 ^$^	83.2 ± 4.1 ^$^	45.9 ± 2.1 ^$^	83.7 ± 4.9 ^$^	51.3 ± 3.4 ^$^
CCFW	96.2 ± 3.9	95.8 ± 3.1	72.6 ± 1.5	75.4 ± 2.4	196.9 ± 3.6	337.1 ± 7.7	31.5 ± 2.3	29.7 ± 0.7	272.0 ± 8.1	117.7 ± 7.5
CCLM	28.1 ± 0.9 ^$^	23.9 ± 1.3 ^$^	20.9 ± 0.6 ^$^	22.7 ± 0.7	25.9 ± 1.4 ^$^	28.6 ± 1.5 ^$^	20.9 ± 0.9 ^$^	17.0 ± 0.9 ^$^	63.4 ± 3.6 ^$^	49.4 ± 1.8 ^$^
CCLW	57.5 ± 3.1	54.6 ± 0.9	95.8 ± 2.5	47.8 ± 0.2	185.0 ± 5.8	122.8 ± 2.0	96.3 ± 3.6	59.3 ± 3.6	149.3 ± 4.6	158.7 ± 13.1

Values, calculated based on the results of the MTT assay, presented as mean ± SE. ^$^ *p* < 0.05, Student’s *t*-test compared to corresponding water extract.

**Table 5:** Selectivity indexes of *C. coggygria* extracts.

Extract	Cal 27		FADU		SW480		HCT116	
	48 h	72 h	48 h	72 h	48 h	72 h	48 h	72 h
CCBM	1.33	1.18	1.03	1.93	0.87	7	2.68	2.60
CCBW	/	/	/	/	/	/	**/**	/
CCFM	2.51	1.25	2.26	2.11	0.62	1.55	1.01	1.12
CCFW	2.83	1.23	3.75	1.56	1.38	0.35	8.63	3.96
CCLM	2.26	2.07	3.03	2.18	2.45	1.73	3.03	2.91
CCLW	2.60	2.91	1.56	3.32	0.81	1.29	1.55	2.68

The selectivity index of each *C. coggygria* extract was calculated using the formula: Selectivity index = IC_50_ MRC-5/IC_50_ tumor cells. /—not applicable.

## Data Availability

The raw data supporting the conclusions of this article will be made available by the authors on request.

## Supplementary Materials

The following supporting information can be downloaded at: https://www.mdpi.com/article/10.3390/ph18010098/s1, Figure S1: UV spectra of compounds identified in *Cotinus* extracts; Figure S2: Chromatogram of *C. coggygria* water leaf extract recorded at (a) 280 nm and (b) 350 nm; Figure S3: Chromatogram of *C. coggygria* water flower extract recorded at (a) 280 nm and (b) 350 nm; Figure S4: Chromatogram of *C. coggygria* water bark extract recorded at (a) 280 nm and (b) 350 nm.
