# Supplementary material for: New Evidence for Cotinus coggygria Scop. Extracts Application in Gastrointestinal Ailments
Source: Pharmaceuticals (Basel). 2025 Jan 15;18(1):98. doi: 10.3390/ph18010098 (PMC11769289; doi:10.3390/ph18010098)
Supplement: Supplementary file 1 [file pharmaceuticals-18-00098-s001.zip › pharmaceuticals-3371929-supplementary.pdf]

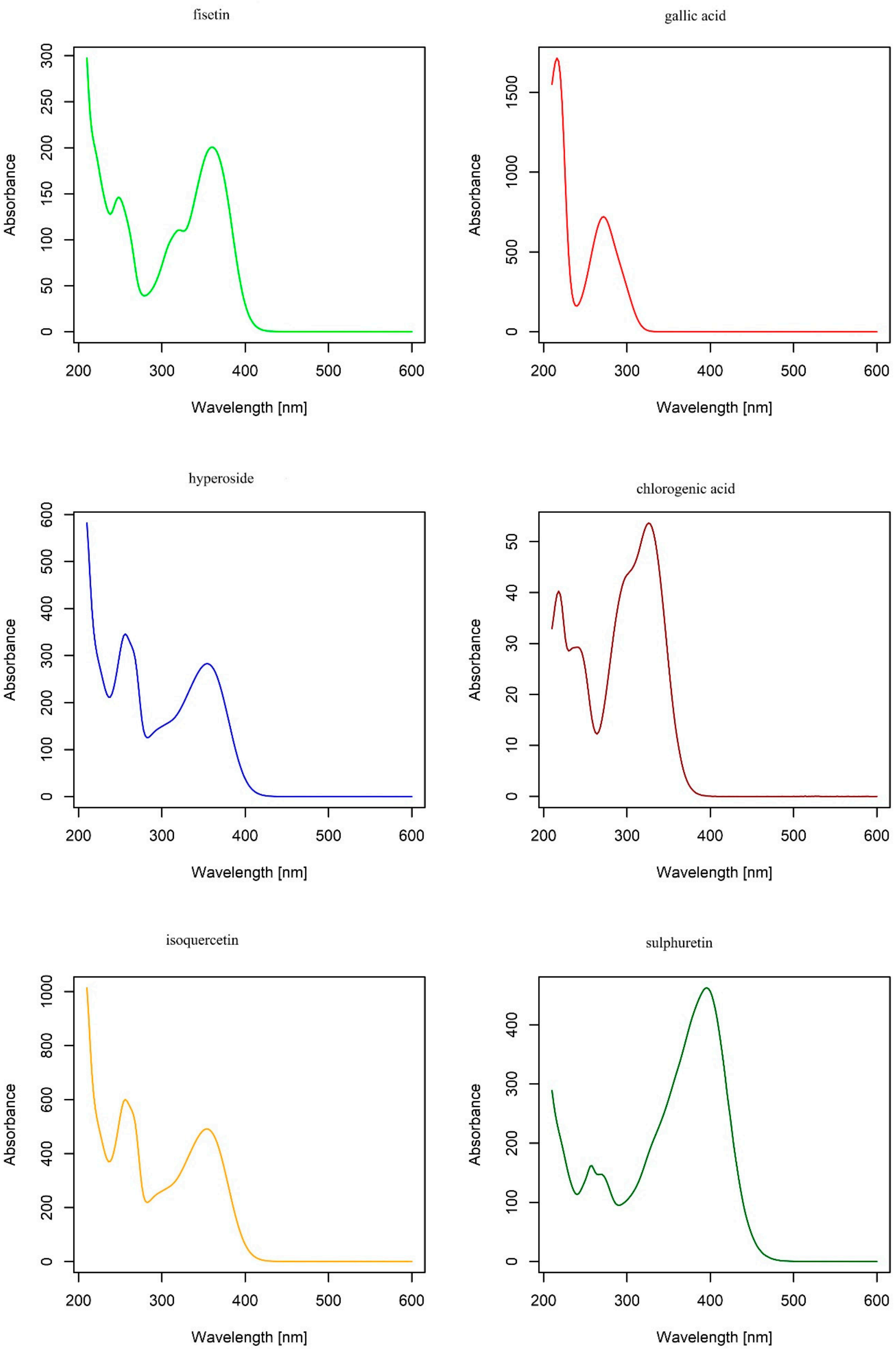

Figure S1. UV spectra of compounds identified in *Cotinus* extracts

a)

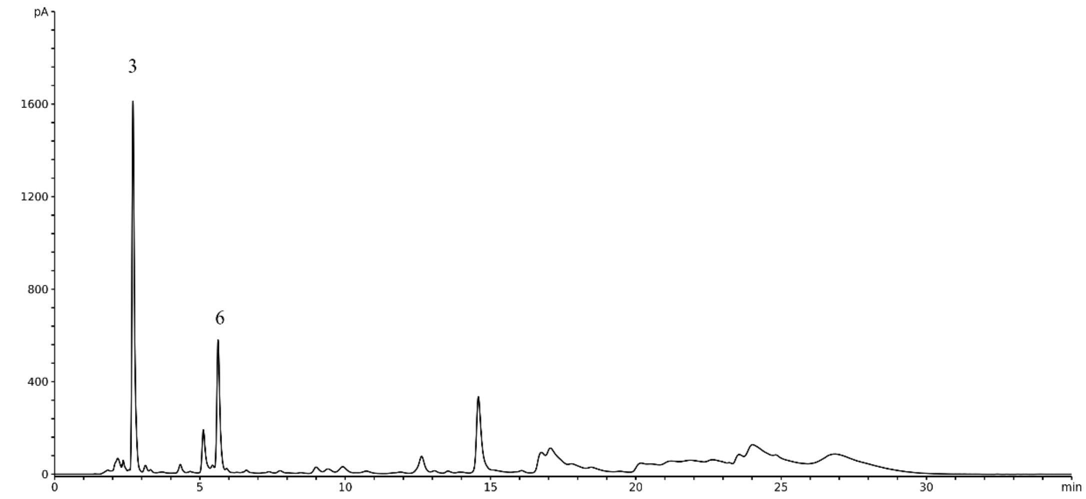

b)

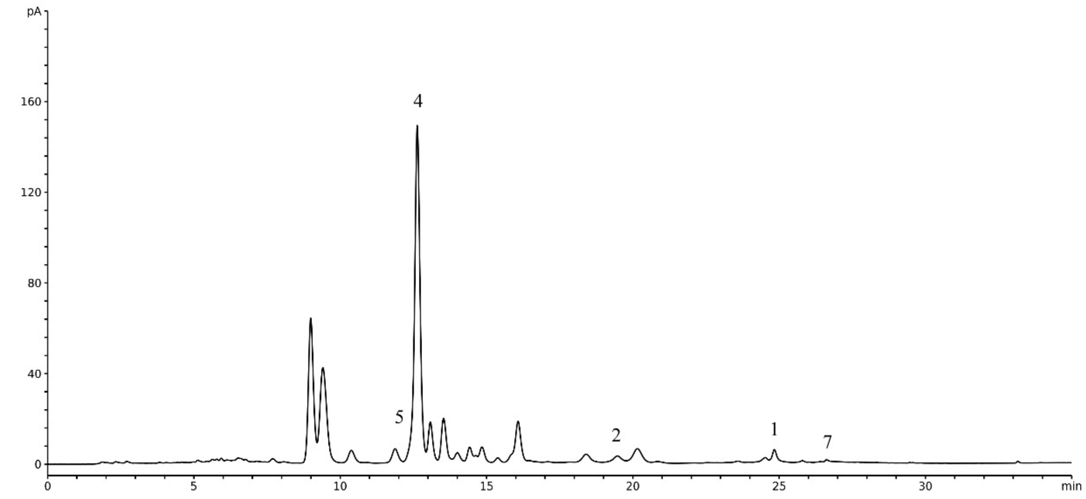

Figure S2. Chromatogram of *C. coggygria* water leaf extract recorded at a) 280 nm and b) 350 nm. 1 - sulphuretin, 2 – fisetin, 3 – gallic acid, 4 – isoqercetin, 5 – hyperoside, 6 – chlorogenic acid, 7 – quercetin.

a)

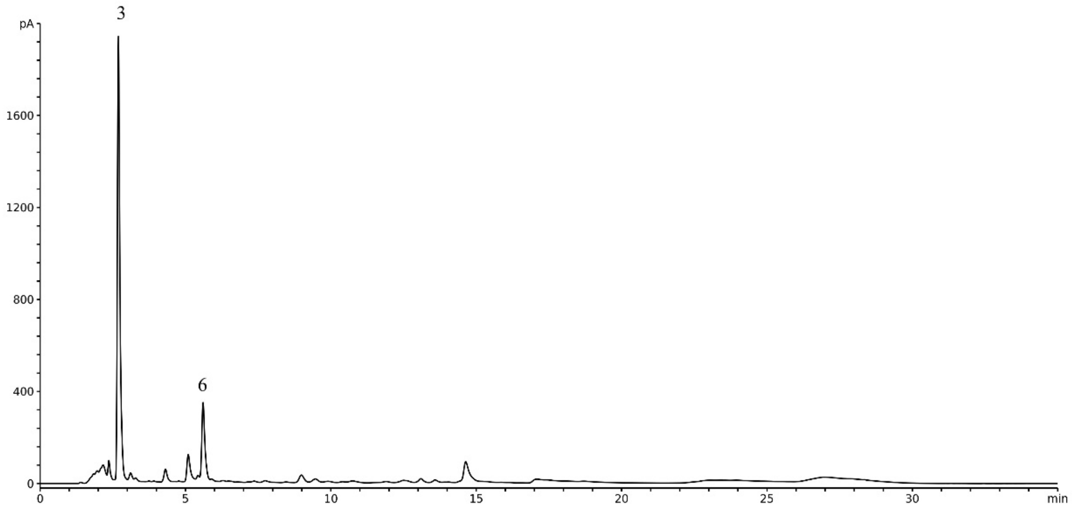

b)

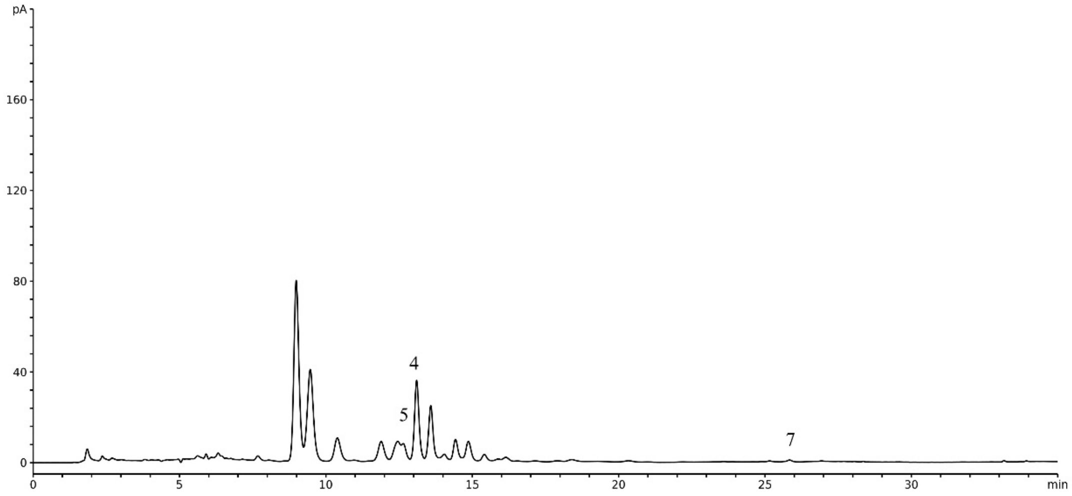

Figure S3. Chromatogram of *C. coggygria* water flower extract recorded at a) 280 nm and b) 350 nm. 1 - sulphuretin, 2 – fisetin, 3 – gallic acid, 4 – isoqercetin, 5 – hyperoside, 6 – chlorogenic acid, 7 – quercetin.

a)

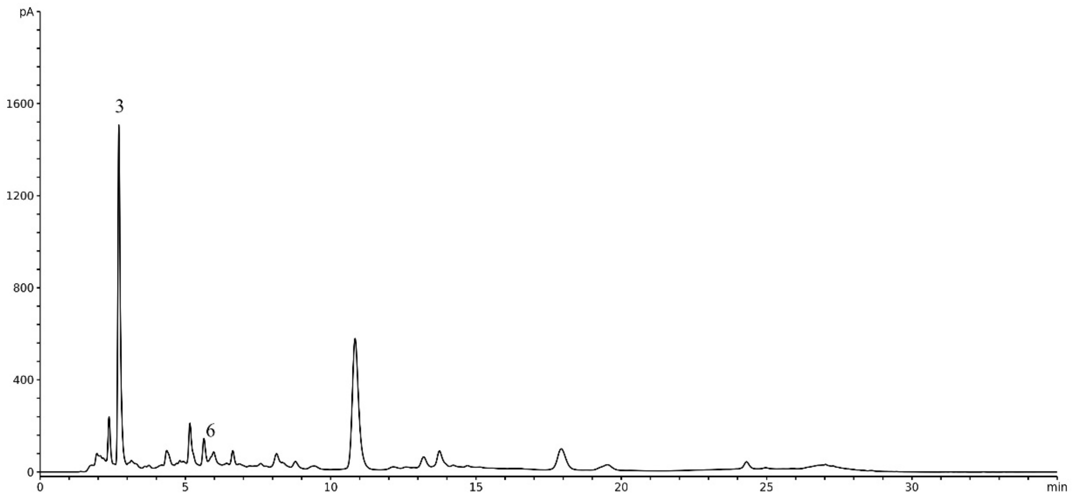

b)

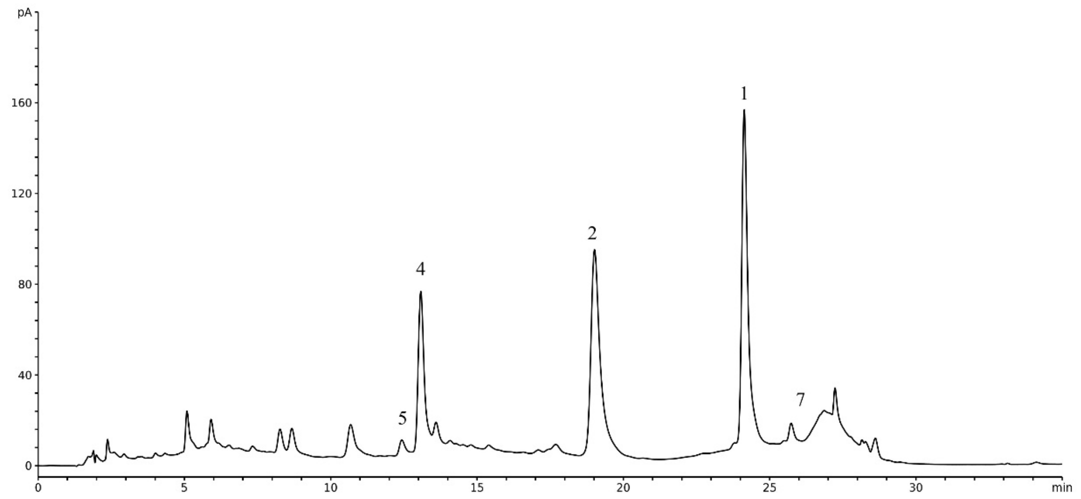

Figure S4. Chromatogram of *C. coggygria* water bark extract recorded at a) 280 nm and b) 350 nm. 1 - sulphuretin, 2 – fisetin, 3 – gallic acid, 4 – isoqercetin, 5 – hyperoside, 6 – chlorogenic acid, 7 – quercetin.
